# Supplementary material for: AEGIS: an annotation extraction and genomic integration resource
Source: Bioinformatics. 2026 Jun 9;42(6):btag363. doi: 10.1093/bioinformatics/btag363 (PMC13296998; doi:10.1093/bioinformatics/btag363)
Supplement: btag363_Supplementary_Data [file btag363_supplementary_data.zip › supp_mat/AEGIS MS - Supplementaries.docx]

AEGIS: an annotation extraction and genomic integration resource

David Navarro-Payá^1,+,*^, Antonio Santiago^1,2,+^, Amandine Velt^3^, Marco Moretto^4^, Camille Rustenholz^3^, and José Tomás Matus^1,*^

^1^Institute for Integrative Systems Biology (I^2^SysBio, UV-CSIC), Valencia, Spain, ^2^Programa de Doctorado en Biotecnología, Universitat Politècnica de València, ^3^INRAE-Université de Strasbourg, SVQV, 68000 Colmar, France, ^4^Unit of Digital Agriculture, Research and Innovation Centre, Fondazione Edmund Mach Via E. Mach 1, 38098 San Michele all'Adige, Italy

+Equally contributed to this work.

**^*To whom correspondence should be addressed.^**

# Supplementary Information

#
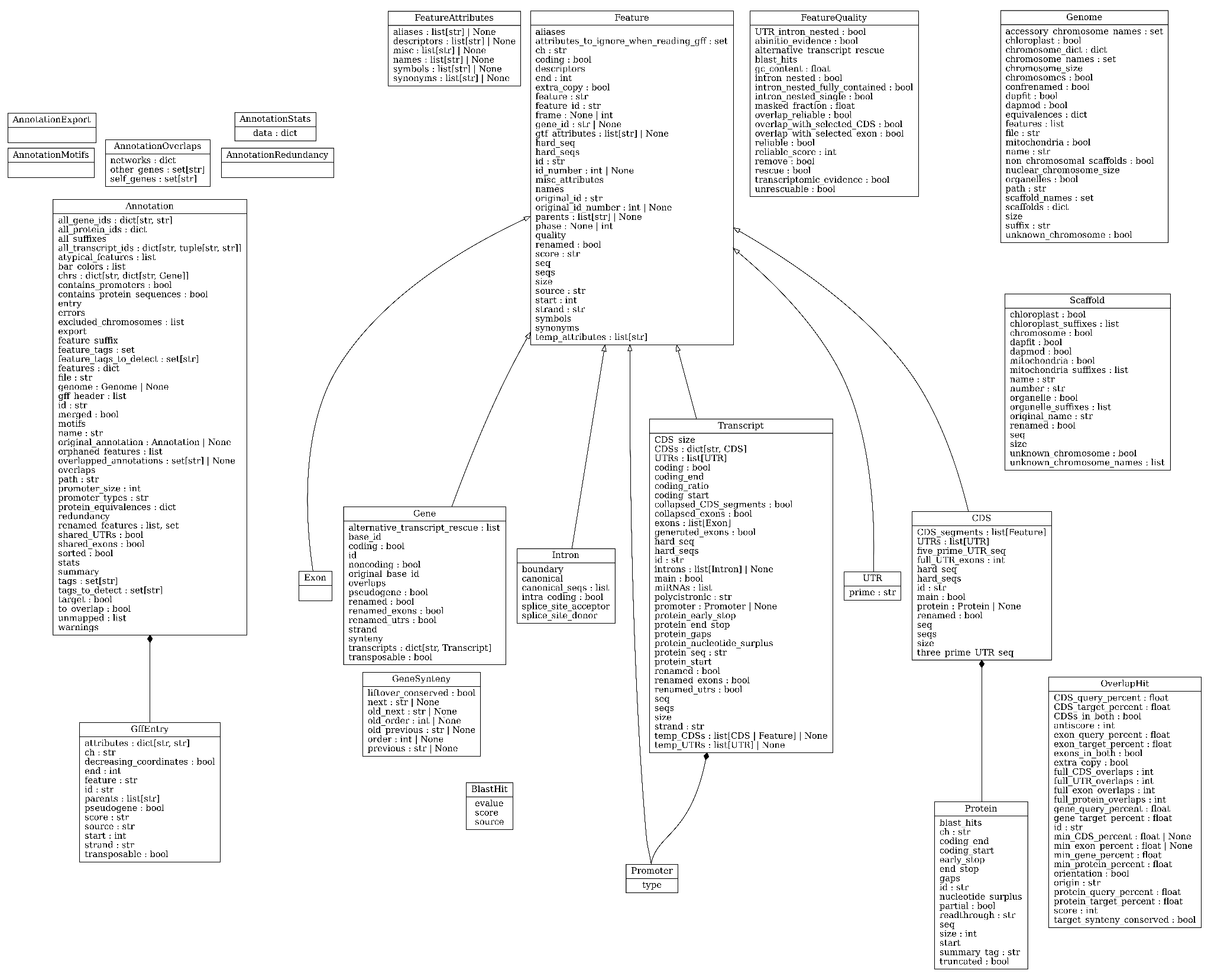


Supplementary Figure S1: UML diagram of AEGIS Python classes and their attributes. Custom classes have been made for each annotation feature and wherever useful inheritance has been used to ensure code efficiency and reliability. All of the classes have been exposed at the top level of the package so they can be directly imported in Python, i.e. ‘*from aegis import Annotation*’ without having to know the specific module where the class is located. A detailed UML diagram including class methods can be found in the package’s main page (<https://github.com/Tomsbiolab/aegis>).


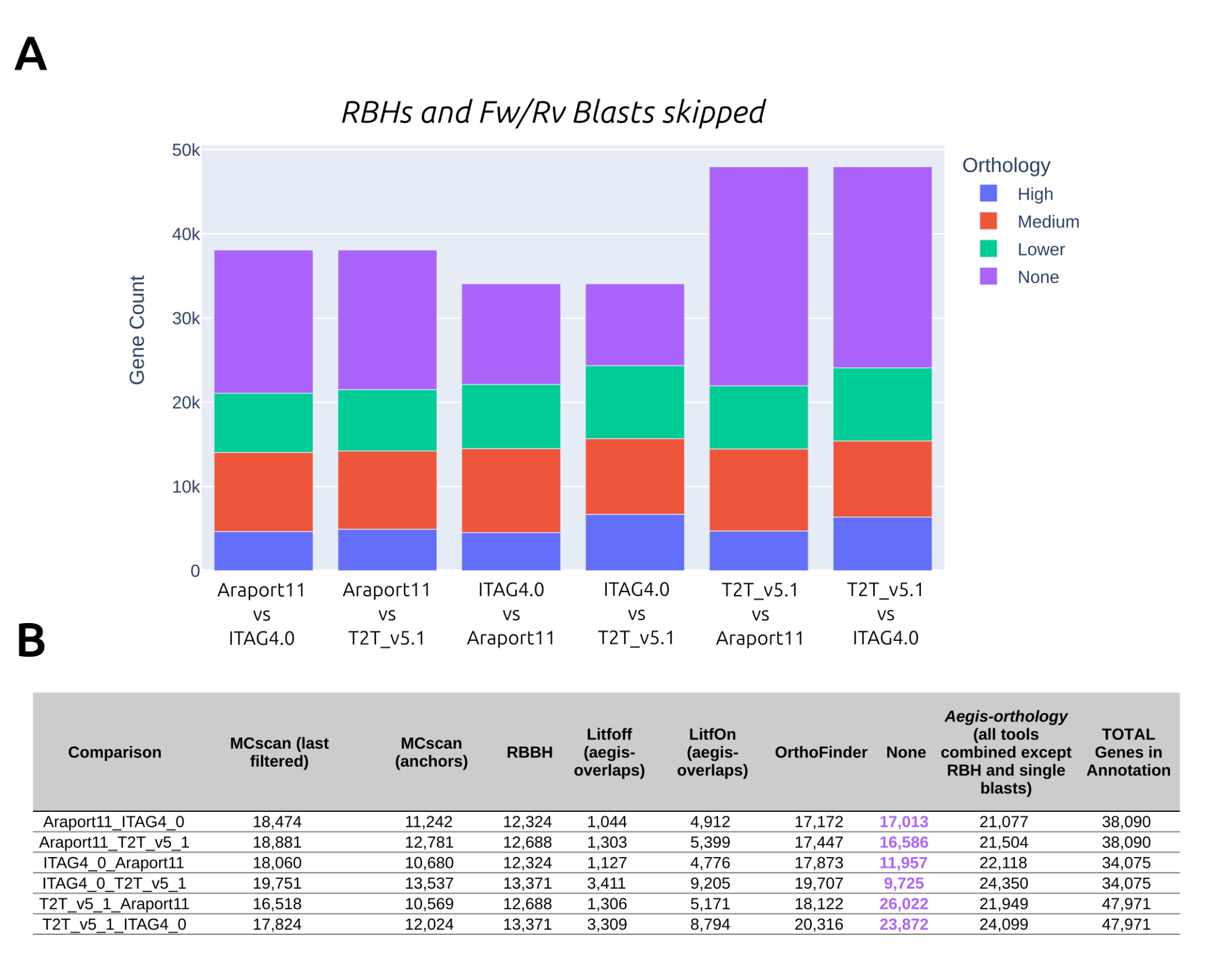


Supplementary Figure S2. Results of *orthology* tool skipping secondary reciprocal and single forward or reverse BLAST hits. The *orthology* tool can be run with the ‘*--skip_rbhs*’ option to obtain a smaller output table that nevertheless still manages to detect orthologues for many genes. The exact output of this *orthology* is included in Supplementary Table S4. (A) The number of genes with no detected orthologues is greater in this case than with the default options (Figure 5A). (B) The combined results of *orthology* report more genes with at least one orthologue when compared to individual tool results.


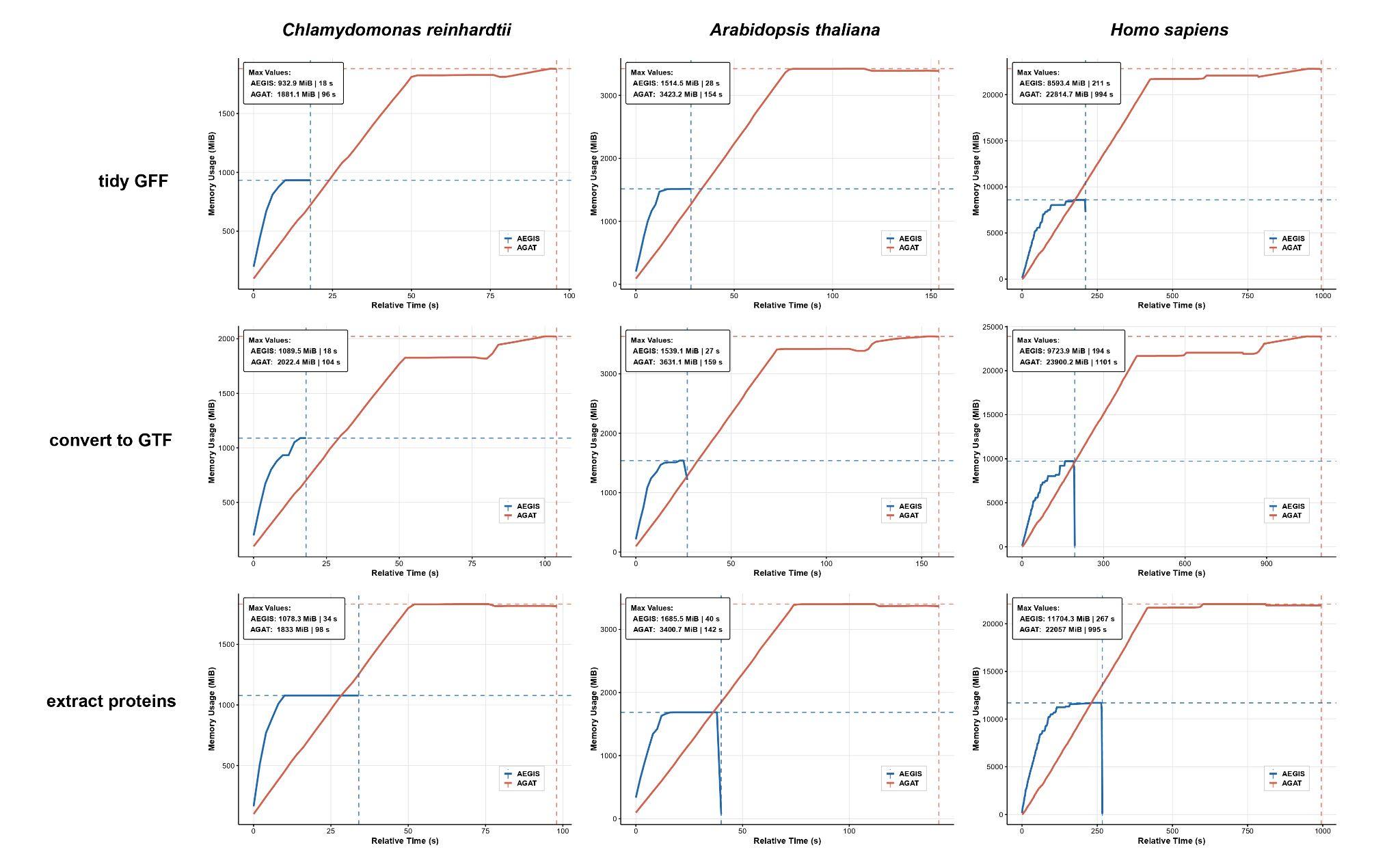


**Supplementary Figure S3**. Computational performance benchmark of AEGIS and AGAT. Memory usage (MB) and relative execution time (s) were compared across three representative genomes: *Chlamydomonas reinhardtii* (small), *Arabidopsis thaliana* (medium), and *Homo sapiens* (large). Performance was evaluated for three core tasks: (top) GFF tidying (AEGIS ***tidy*** vs. AGAT agat_convert_sp_gxf2gxf.pl), (middle) GTF conversion (AEGIS ***reformat*** vs. AGAT agat_convert_sp_gff2gtf.pl), and (bottom) protein extraction (AEGIS ***extract*** vs. AGAT agat_sp_extract_sequences.pl).
